# A rare case report of gemcitabine-induced thrombotic microangiopathies

**DOI:** 10.1177/2050313X211013208

**Published:** 2021-05-24

**Authors:** Mohammed Ali Madkhali

**Affiliations:** Division of Hematology, Hemostasis, Oncology and Stem Cell Transplantation, Department of Internal Medicine, Hannover Medical School, Hannover, Germany

**Keywords:** Case report, gemcitabine, thrombotic microangiopathies

## Abstract

A 65-year-old female patient with breast cancer and soft tissue sarcoma who experienced a gemcitabine-induced thrombotic microangiopathies manifestation visited the emergency department. The renal biopsy revealed endothelial cell swelling, focal reduplication of glomerular basement membrane, and narrowed capillary lumens with fibrin deposition and fragmented erythrocytes which are compatible with thrombotic microangiopathies. The patient was presented with organ injury, acute renal failure with the need for hemodialysis technique. The patient recovered after the appropriate treatment. Continuous observation of renal function during gemcitabine treatment regularly and withdrawal of treatment if acute kidney injury occurs is essential as acute kidney injury along with thrombotic microangiopathies may prove to be life-threatening.

## Introduction

Thrombotic microangiopathies (TMAs) are microvascular occlusive diseases caused by mechanical damage of erythrocytes and platelet aggregation. The disorder is characterized by thrombocytopenia, microangiopathic hemolytic anemia, and different grades of organs injuries (cardiac, neurological, and acute renal failure) resulting from systemic microvascular thrombi. Primary TMA diseases are classified into (1) thrombotic thrombocytopenic purpura (TTP), in their acquired and congenital (Upshaw–Schulman syndrome) types, and (2) hemolytic–uremic syndrome (HUS), related to infection caused by Shiga toxin–producing infectious agent and the atypical HUS related to uncontrolled alternative pathways activation of complement activation. The secondary TMAs are associated with severe preeclampsia in pregnant women and HELLP (hemolysis, elevated liver enzymes, low platelet count) syndrome. It is also induced by the autoimmune disease, systemic infections (H1N1, HIV), organ transplantations, and some drugs. The TMA incidence in cancer patients is rare; however, it has severe consequences.^1^

The TMA in cancer patients is caused by two major reasons. First, the cancer itself may cause TMA by bone marrow or systemic microvascular metastases and second using common oncotherapy drugs. Chemotherapy may induce TMA either by dose-dependent toxicity or acute immune-mediated reaction. Gemcitabine, a chemotherapy drug, is a nucleoside analogue which is used against several types of cancers such as ovarian, breast, pancreatic, bladder, sarcoma, and non-small-cell lung cancer. However, gemcitabine has several major side effects such as myelosuppression, respiratory failure, mild liver dysfunction, elevated blood pressure, and gastroenterological symptoms. The incidence of gemcitabine-induced TMA ranges between 0.015% and 0.30%. The majority patients develop TMA between 1 and 2 months of last infusion.^2,3^ At present no standard guidelines are available for managing gemcitabine-induced TMA.

## Case

A 65-year-old female patient was presented at the emergency center of Hannover Medical School in November 2019 with the symptoms of increasing bilateral leg swelling, dizziness, fatigue, and dyspnea. The patient had a history of metastatic soft tissue sarcoma and breast cancer. In October 2016, the patient was first diagnosed with pleomorphic sarcoma (G3) in the left gluteal region with lung metastasis. The primary tumor and the metastases were removed followed by adjuvant radiation therapy. From March 2019, she was treated with doxorubicin and bevacizumab for relapsing metastatic soft tissue sarcoma. The patient was treated for five treatment cycles (approximately 4 months) followed by computed tomographic (CT) scan in July 2019. The patient was presented with mucositis and severe hematotoxicity. Therefore, and due to disease progression, the treatment regime was changed to gemcitabine and docetaxel for two cycles with a cumulative dosage of 1800 mg/m^2^ of gemcitabine. In September 2019, cancer staging showed stability of the disease. But due to suspected drug hematotoxicity, the treatment was reduced. The patient continued to receive the therapy in reduced dose for three cycles with a cumulative dose of 2025 mg/m^2^ gemcitabine till October 2019. In addition, in March 2017, the patient was also diagnosed with metastatic breast cancer left breast with lung metastasis. The histology revealed invasive lobular type of breast cancer. In May 2017, mastectomy was performed to remove the tissue. The patient is currently being treated for breast cancer with tamoxifen since September 2018.

During the emergency arrival, the initial assessment of the patient showed the blood pressure as 170/93 mm Hg, temperature as 37.1°C with 95% oxygen saturation while breathing in the ambient air. The physical examination showed peripheral edema and weakened breath sounds bilaterally. The laboratory examination revealed the platelet count as 88 × 103 μL, hemoglobin as 5.9 g/dL, and leucocytes count of 12 × 103 μL. Serum creatinine level was 472 μmol/L, serum potassium was 4.4 mmol/L, and serum sodium was 119 mmol/L. The patient was admitted due to severe anemia and acute renal failure and thus was treated with fluids and blood transfusion. Blood test investigation was done for anemia studies which showed the hemoglobin level as 5.9 g/dL (post transfusion hemoglobin 7.9 g/dL), haptoglobin < 0.1 g/L, and lactate dehydrogenase level as 1510 U/L. Furthermore, the negative direct antiglobulin test and high schistocytes count on blood smear were diagnosed as microangiopathic hemolytic anemia. The microangiopathic hemolytic anemia, thrombocytopenia, and anuric renal failure thus led to TMA diagnosis. Also, there was no diarrhea or any other symptoms suggesting any infection. The antinuclear antibody (ANA), extractable nuclear antigen (ENA), C3, C4 test for diagnosing any autoimmune conditions were found to be within the normal level. After 24 h, the ADAMTS13 test results showed normal range, thus diagnosis of TTP was ruled out.

After diagnosis, treatment with methylprednisolone with 1 mg/kg dose per day was given to the patient. Renal biopsy confirmed the diagnosis along with histological alterations compatible with involvement of TMA at initial acute phase. CT scan and bone marrow biopsy done were also done during the hospital stay to rule out any disease progression or bone marrow infiltration being cancer cells. According to the CT finding and bone marrow biopsy, there was no significant tumor progression or no bone marrow infiltration, respectively. Regardless of well-established treatment, the patient showed signs of anuria with unfavorable blood test results. Anuria conditions led to first session of hemodialysis. The anemic state was increased at a slow rate; therefore, red-cell transfusion was not performed after third day of admission. There was no need of transfusion for the platelet count. The patient showed progressive improvement of the symptoms after the hemodialysis. Renal failure of the patient showed slow improvement, with recovery of diuresis on third week and requirement of hemodialysis session for 6 weeks. The major laboratory findings are shown in Table 1. The patient was discharged after 28 days of admission. The timeline of the patient cancer history and TMA treatment is presented in Supplementary Table 1.

**Table 1.** Laboratory findings of the patient.

Date of test	20.09.2019	04.10.2019	24.10.2019	01.11.2019	07.11.2019	09.11.2019	14.11.2019	21.11.2019
Leucocytes (Tsd/µL)	6.6	2.3	1.4	8.4	12.2	9.5	10.5	15.3
Hemoglobin (g/dL)	9.1	8.6	6.2	7.8	5.9	9.4	9.2	8.2
Platelet (Tsd/µL)	453	315	61	65	82	95	147	143
Lactate dehydrogenase (LDH) (U/L)	678	804	1168	1320	1510	936	650	447
Haptoglobin (g/L)	–	–	–	–	<0.1	–	–	0.58
Creatinine (µmol/L)	109	127	204	255	472	481	614	353

## Discussion

The detection of unexpected TMA which is characterized in cancer patient by thrombocytopenia, microangiopathic hemolytic anemia, and ischemic end-organ damage warrants an urgent diagnosis and appropriate patient management. The drug-induced secondary TMA is caused by chemotherapeutic agents such as gemcitabine, mitomycin C, and cisplatin.^1^ In our present case report, the female patient with history of malignancy was treated using gemcitabine, which is compatible with the secondary TMA. The TMA in the patient may have been triggered or complicated due to some other glomerular condition, such as focal segmental glomerulosclerosis and membranous nephropathy. Second, there was rapid increase in the Adenylate kinase isoenzyme1 (AKI) of serum creatinine level of the patient. Rapid progress in AKI may cause fatal organ failure and irreparable end-stage renal disease. Due to these conclusions, renal biopsy was done in the presented patient. The findings from renal biopsy specimen were compatible with the TMA.

Gemcitabine can cause direct injuries in the endothelium and thus may lead to TMA in cancer patients. Till date, the comprehensive mechanism of drug-induced TMA is not known, but prostacyclin or prostaglandin I2 is thought to decrease von Willebrand factor secretion, with endothelial cell injury and subsequent aggregation of platelet. TMA is generally induced after 5–8 months post treatment. One of the most common clinical symptoms reported post-gemcitabine treatment of TMA is newly developed hypertension.^3,4^ In the present case, the patient also showed the elevated blood pressure. Gemcitabine-induced TMA is treated by its discontinuation, dialysis, and anti-hypertensive therapy. Generally, it is enough to eliminate the TMA-inducing drug from the treatment protocol for resolving renal and hematological issues.^3^ In the present case, patient showed significant improvement after 2 months of gemcitabine cessation. The therapeutic options with plasmapherese or eculizumab have a limited success in the treatment of gemcitabine-induced TMA. Similar cases of gemcitabine-induced TMA in three different patients with the symptoms of chronic renal impairment, shock, hemolytic anemia, and other symptoms were previously reported by a retrospective study. These patients were also treated by ceasing the use of gemcitabine and performing dialysis with other required treatment. The renal functionality slowly recovered in these patients. But two patients eventually died due to other severe complications of TMA and one patient survived after intensive care. Thus, early diagnosis of TMA and intensive care of patients with drug-induced TMA are significant in cancer patients.^5^

After weighing up the risk–benefit ratio and due to poor prognosis of the patient, we treated her conservatively. Despite the established treatment, the persistent anuria condition led to perform first hemodialysis session. At the hematological level, anemia increased very gradually; thus, red-cell transfusion was not required after the third day of admission. After the hemodialysis, the patient showed progressive improvement in the symptoms. Renal failure was slowly improved, and diuresis was recovered in the second week. The hemodialysis session was required till the last day of hospital stay. Despite metastases and palliative situation, the dialysis for 6 weeks led to recovery of renal function. As TMA was diagnosed comparatively early in the present patient, she was able to recover and thus improving her quality of life as compared to above-reported similar patients who died due TMA complications. After the improvement of renal function and due to the patient request, hemodialysis was discontinued after 6 weeks. The chemotherapy was changed to palliative therapy with pazopanib.

## Conclusion

Timely diagnosis of drug-induced toxicity and TMA is important for proper metastatic cancer treatment and to evade probable drug toxicity.

## Supplemental Material

sj-docx-1-sco-10.1177_2050313X211013208 – Supplemental material for A rare case report of gemcitabine-induced thrombotic microangiopathies Click here for additional data file. Supplemental material, sj-docx-1-sco-10.1177_2050313X211013208 for A rare case report of gemcitabine-induced thrombotic microangiopathies by Mohammed Ali Madkhali in SAGE Open Medical Case Reports
