# Supplementary material for: A rare case report of gemcitabine-induced thrombotic microangiopathies
Source: SAGE Open Med Case Rep. 2021 May 24;9:2050313X211013208. doi: 10.1177/2050313X211013208 (PMC8155781; doi:10.1177/2050313X211013208)
Supplement: sj-docx-1-sco-10.1177_2050313X211013208 – Supplemental material for A rare case report of gemcitabine-induced thrombotic microangiopathies [file sj-docx-1-sco-10.1177_2050313X211013208.docx]

**Supplementary Table 1: Timeline of the case**

| **Date** | **Timeline for Soft tissue**  **sarcoma disease** | **Treatment/ Result** |
| --- | --- | --- |
| October 2016 | First diagnosis of metastatic soft tissue sarcoma in left gluteal region with lung metastasis | Resection of the primary Tumor and Metastasectomy |
| November 2016- January 2017 | - | Adjuvant radiation therapy |
| March- June 2019 | - | Doxrubicin and bevacizumab |
| July 2019 | CT to check progress | Gemcitabine and docetaxel due to Progress and toxicity |
| September 2019 | Staging | Disease Stable.  Dose reduction due to hematotoxicity |
|  | **Timeline for Metastatic breast cancer** |  |
| March 2017 | First diagnosis of breast cancer in left breast with lung metastasis | - |
| May 2017 | - | Mastectomy performed |
| September 2018 | - | Treatment with Tamoxifen |
| 10 October 2019 |  | Gemcitabine treatment was stopped |
|  | **Timeline for Gemcitabine-induced TMA** |  |
| 07 November 2019 | Patient presented in emergency department | - |
| 07 November 2019 | Initial assessment | Admitted due to severe anemia and acute renal failure. Treated with fluids and blood transfusion |
|  | Direct antiglobulin test  blood smear | Negative antiglobulin test and high schistocytes count.  Diagnosed as microangiopathic haemolytic anemia. |
|  | Microangiopathic haemolytic anemia, thrombocytopenia and anuric renal failure | TMA diagnosis |
| 07 November 2019 | ADAMTS13 test | TTP was ruled out |
| 08 November 2019 | Prognostic CT and bone marrow | No significant tumor progression or bone marrow infiltration found |
| 09 November 2019 | Treatment with methylprednisolone started | 1 mg/kg dose per day |
| 12 November 2019 | Renal biopsy | Confirmed TMA diagnosis |
| 15 November 2019 | Haemodialysis due to Anuria | Progressive improvement of symptoms |
|  | Renal failure of patient showed slow improvement | Required hemodialysis session for six weeks |
| 27 November 2019 | Patient discharged | Follow up in the outpatient clinic |
